# CDK4/6 inhibitors versus weekly paclitaxel for treatment of ER+/HER2- advanced breast cancer with impending or established visceral crisis

**DOI:** 10.1007/s10549-023-07035-6

**Published:** 2023-08-16

**Authors:** Roya Behrouzi, Anne C. Armstrong, Sacha J. Howell

**Affiliations:** 1 Department of Medical Oncology, The Christie NHS Foundation Trust, Wilmslow Road, Manchester, M20 4BX UK; 2 Division of Cancer Sciences, The University of Manchester, Manchester, UK

**Keywords:** Advanced breast cancer, ER positive, Visceral crisis, CDK4/6 inhibitor, Paclitaxel, Chemotherapy

## Abstract

**Purpose:**

ER+/HER2- advanced breast cancer (ABC) with visceral crisis (VC) or impending VC (IVC) is commonly treated with chemotherapy instead of CDK4/6 inhibitors (CDK4/6i). However, there is little evidence to confirm which treatment is superior. This study compared outcomes of patients with ER+/HER2- ABC and IVC/VC treated with CDK4/6i or weekly paclitaxel.

**Methods:**

Patients with ER+/HER2- ABC receiving first line treatment at a large tertiary UK cancer centre from 1-Mar-2017 to 30-Jun-2021 were retrospectively identified. Hospital records were screened for IVC/VC affecting the liver, lungs/mediastinum, gastrointestinal tract and/or bone marrow. Baseline demographics, clinical data and survival outcomes were recorded up to 30-Jul-2022.

**Results:**

27/396 (6.8%) patients with ABC who received CDK4/6i and 32/86 (37.2%) who received paclitaxel had IVC/VC. Median time to treatment failure (TTF), progression-free survival (PFS) and overall survival (OS) were significantly longer in the CDK4/6i compared to paclitaxel cohort: TTF 17.3 vs. 3.5 months (HR 0.33, 95%CI 0.17–0.61, *p* = 0.0002), PFS 17.8 vs. 4.5 months (HR 0.38, 95%CI 0.21–0.67, *p* = 0.002), OS 24.6 vs. 6.7 months (HR 0.37, 95%CI 0.20–0.68, *p* = 0.002). The median time to first improvement in IVC/VC was similar in patients receiving CDK4/6i compared to paclitaxel (3.9 vs. 3.6 weeks, *p* = 0.773). Disease control at 4 months was not significantly different in the CDK4/6i and paclitaxel cohorts (77.8% vs. 59.4%, *p* = 0.168). In multivariate analysis, treatment with CDK4/6i was independently associated with a longer PFS compared to paclitaxel (HR 0.31, 95%CI 0.12–0.78, *p* = 0.015).

**Conclusion:**

In this retrospective study, patients with ER+/HER2- ABC and IVC/VC treated with CDK4/6i had a significantly better survival compared to those treated with weekly paclitaxel. Further prospective studies that minimise possible selection bias are recommended.

## Introduction

Systemic therapy for ER+/HER2- ABC has changed significantly over the last 10 years with the introduction of CDK4/6i. All three licenced CDK4/6i (ribociclib, palbociclib and abemaciclib) in combination with endocrine therapy (ET) have been shown to markedly improve PFS, and in some cases OS, compared to ET alone [1–3]. This has led to an increased use of CDK4/6i and a decline in chemotherapy for first line treatment of ABC. Visceral metastases are associated with lower response rates to systemic therapies including CDK4/6i + ET and a poorer prognosis [4, 5]. However, international guidelines recommend CDK4/6i + ET as the optimal first line therapy for ER+/HER2- ABC with or without visceral metastases [6]. The terms visceral crisis (VC) and impending visceral crisis (IVC) have been introduced in an attempt to describe the degree of organ dysfunction caused by metastases in ABC. VC is defined by ESO-ESMO ABC international guidelines as metastases causing “severe organ dysfunction, as assessed by signs and symptoms, laboratory studies and rapid progression of disease” [6]. It is estimated to affect 10–15% of patients with ABC receiving first line treatment and is associated with a very poor prognosis [7]. IVC is the clinical situation where criteria for VC are not met but organ compromise is believed to be imminent based on clinical, biochemical and/or radiological parameters, which is more subjective. In patients with IVC/VC, “rapidly efficacious therapy” is recommended [6] although the most efficacious therapy in this setting is not known and, traditionally, chemotherapy is frequently used. In a study of carboplatin-based combination chemotherapy in patients with ABC (58% ER+/HER2-, 12% first line), patients with VC had a response rate of 27% and median OS of 3.7 months [8]. In a retrospective study of 44 patients with ABC and VC that received weekly paclitaxel + bevacizumab (80% ER+/HER2-, 68% first line), the response rate was 41% and median OS was 10.8 months [9]. Two recent retrospective studies have reported outcomes of CDK4/6i in patients with VC [10, 11]. The first was in patients with ER+/HER2- ABC, of whom 336 (5.6%) had VC; patients that received a CDK4/6i + ET had a median OS of 11 months compared to 6 months in those that received any other therapy first line [10]. The second study was of patients with ABC of different receptor subtypes who had VC, and included 92 patients with ER+/HER2- ABC. Patients that received either aromatase inhibitors alone or a CDK4/6i + ET had a longer median OS of 24.3 months compared to only 6.2 months in patients who received chemotherapy [11]. These studies suggest that CDK4/6i could be an efficacious treatment for patients with IVC/VC and produce better survival outcomes compared to chemotherapy.

The aim of this study was to compare the treatment outcomes of patients with IVC/VC commencing first line therapy for ER+/HER2- ABC with CDK4/6i or weekly paclitaxel at our institution since the widespread adoption of CDK4/6i.

## Methods

### Patient selection

**Fig. 1:**
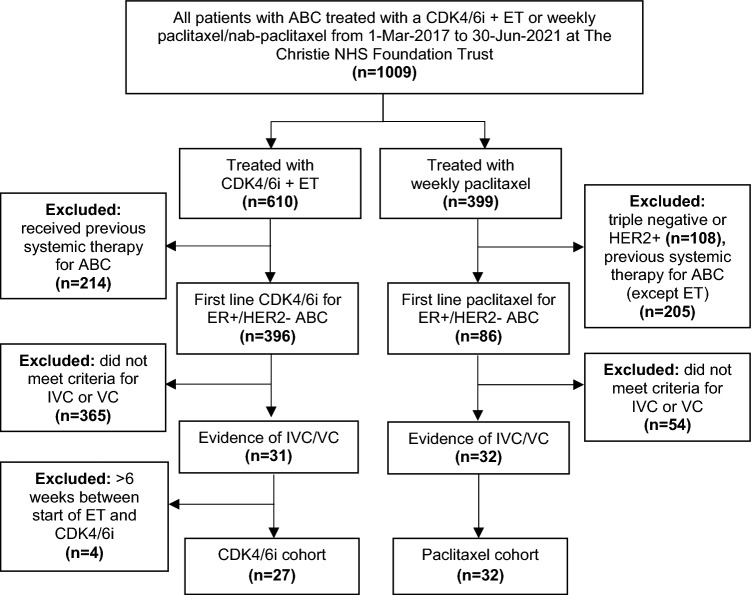
CONSORT diagram for retrospective selection of study patients

Patients with ER+/HER2- ABC who commenced first line treatment with a CDK4/6i + ET or weekly paclitaxel/nab-paclitaxel between 1-Mar-2017 and 30-Jun-2021 at The Christie NHS Foundation Trust, a large UK tertiary cancer centre, were identified from electronic medical and prescription records. The standard weekly regimen was 80 mg/m^2^ for paclitaxel and 100 mg/m^2^ for nab-paclitaxel and any dose reductions were carried out according to local protocols. According to local guidelines, nab-paclitaxel was used only if paclitaxel was contraindicated due to previous taxane reactions. Figure 1 shows the CONSORT diagram for the process of subject identification. Patients were excluded if they had received previous CDK4/6i or any other systemic anti-cancer therapy for ABC, or commenced ET more than 6 weeks prior to the addition of a CDK4/6i. Previous single-agent ET for ABC was allowed in the paclitaxel cohort. Patients with IVC/VC were selected if they met any of the criteria in Table 1, which were defined based on the descriptive definitions of VC and IVC provided in the ESO-ESMO ABC guidelines [6].

**Table 1:** Criteria for identification of patients with IVC or VC

Organ	Impending visceral crisis (IVC)	Visceral crisis (VC)
Liver	Extensive liver metastases (both right and left lobes) + AST/ALT or bilirubin ≥ 1.2 × upper limit of normal (ULN), in the absence of biliary obstruction or non-malignant liver disease	Extensive liver metastases (both right and left lobes) + AST/ALT ≥ 3 × ULN and/or bilirubin ≥ 1.5 × ULN, in the absence of biliary obstruction or non-malignant liver disease
Lung/mediastinum	Bilateral lung metastases with significant dyspnoea on mild exertion, or impending superior vena cava obstruction (SVCO), in absence of known chronic lung disease	Lymphangitis or bilateral lung metastases with significant dyspnoea at rest or oxygen saturations < 95% on air, or SVCO, in absence of known chronic lung disease
Gastrointestinal	Intra-abdominal/peritoneal metastases with clinical symptoms of bowel obstruction, without radiological evidence of bowel obstruction	Intra-abdominal/peritoneal metastases with radiological evidence of bowel obstruction
Bone marrow	–	Extensive bone metastases and two or more of the following below the lower limit of normal: haemoglobin, neutrophil count and/or platelet count, in the absence of a non-breast cancer associated haematological disorder

### Data collection

Baseline demographics and clinical and physiological parameters were recorded for each patient from ≤ 1 week of the treatment start date. Tumour response at 4 months (after first imaging) was assessed based on retrospective review of radiology images and reports and where possible, in accordance with Response Evaluation Criteria in Solid Tumours (RECIST) version 1.1 criteria of measurable lesions [12]. Disease control at 4 months was defined as partial/complete response or stable disease. ET-sensitivity or resistance was recorded using the following definitions: primary ET-resistance = relapse within 2 years of starting adjuvant ET or progression within 6 months of starting ET for ABC; secondary ET-resistance = relapse > 2 years after starting adjuvant ET but < 12 months after completion of ET, or progression > 6 months after starting ET for ABC; ET-sensitive = relapse > 12 months after finishing adjuvant ET; ET-naive = never received ET for breast cancer. Treatment characteristics were recorded including dosing, adverse events, date of and reason for stopping. Use of maintenance treatment after completion of paclitaxel and second line treatment after progression on first line systemic therapy were recorded. Time to first objective improvement in IVC/VC was defined as the time from start of treatment to first recorded: ≥ 30% improvement from baseline or normalisation of AST/ALT/bilirubin (liver IVC/VC) or haemoglobin/platelets/neutrophil count (bone marrow IVC/VC) without worsening of another parameter, significant improvement in breathing/oxygen saturation (lung/mediastinum IVC/VC), significant improvement in bowel obstruction symptoms (gastrointestinal IVC/VC) or radiological response/stability (any organ IVC/VC). Time to first objective worsening in IVC/VC was defined as the time from start of treatment to first recorded: ≥ 20% worsening from baseline of AST/ALT/bilirubin (liver IVC/VC) or haemoglobin/platelets/neutrophil count (bone marrow IVC/VC), significant worsening of breathing/oxygen saturation (lung/mediastinum), worsened bowel obstruction symptoms (gastrointestinal IVC/VC) or radiological progression (any organ IVC/VC). Date of clinical/radiological progression (resulting in stopping/change of treatment) and date of death were recorded, where applicable. The cut-off date for retrospective follow up was 30-Jul-2022 and follow up was censored at this date for study endpoints.

### Data analysis

All statistical analyses were performed using GraphPad Prism version 9.0 (GraphPad Software, San Diego, CA, USA). A *p* value < 0.05 was considered statistically significant. The following survival outcome definitions were used: TTF = time from start of treatment to discontinuing treatment due to cancer progression, death, treatment toxicity, clinical deterioration, reduced performance status (PS) and/or patient choice (if patients had completed paclitaxel without evidence of cancer progression then it was until progression or death during maintenance treatment or surveillance); PFS = time from start of treatment to radiological/clinical progression or death—whichever occurred sooner; OS = time from start of treatment to date of death. Baseline patient and treatment characteristics were compared between the CDK4/6i and paclitaxel cohorts using Mann-Witney-U tests for continuous data and Fisher's exact or Chi-squared tests for categorical data. Fisher’s exact or Chi-squared tests were used to compare categorical subgroups between cohorts. Log-rank test was used to compare PFS and OS between subgroups. Multivariate analysis was performed using Cox-regression analysis.

## Results

### Baseline patient characteristics

482 patients with ER+/HER2- ABC were identified who had received first line treatment with a CDK4/6i + ET or weekly paclitaxel (Fig. 1). Of these, 59/482 (12.2%) had IVC/VC and met study inclusion criteria—this comprised 27/396 (6.8%) of the CDK4/6i cohort and 32/86 (37.2%) of the paclitaxel cohort. Table 2 shows baseline characteristics of patients in the CDK4/6i and paclitaxel cohorts. The cohorts were generally well balanced for most demographic and disease parameters. However, more patients that received paclitaxel had recurrent rather than de novo disease and more had secondary ET-resistance. Performance status was overall worse in the paclitaxel cohort, mainly due to a greater proportion of PS 2 and smaller proportion of PS 1 patients. In both cohorts, liver was the most common site of IVC/VC and only a minority had more than one organ affected by IVC/VC. A greater proportion of patients treated with paclitaxel met criteria for VC rather than IVC. Table 3 shows baseline blood test results of patients in both cohorts. LDH was significantly higher in patients receiving paclitaxel compared to CDK4/6i. In patients with IVC/VC of the liver, serum AST/ALT was higher and albumin lower in patients who received paclitaxel, but bilirubin was not significantly different.

**Table 2:** Baseline characteristics of patients receiving CDK4/6i or paclitaxel

	CDK4/6i *n* = 27 (%)	Paclitaxel *n* = 32 (%)	*p* value
Age (years)			
Median	59	57	0.589
Range	27–86	39–85	
Menopausal status			
Pre-menopause	6 (22.2)	6 (18.8)	0.485
Peri-menopause/unknown	4 (14.8)	2 (6.3)	
Post-menopause	17 (63.0)	24 (75.0)	
Performance status			
0	6 (22.2)	7 (21.9)	0.037*
1	18 (66.7)	10 (31.3)	
2	2 (7.4)	11 (34.4)	
3	1 (3.7)	3 (9.4)	
4	0 (0)	1 (3.1)	
De novo/recurrence			
De novo	11 (40.7)	2 (6.3)	0.003*
Recurrence	16 (59.3)	30 (93.8)	
Metastatic sites			
Lung/pleura	19 (70.4)	17 (53.1)	0.523
Liver	17 (63.0)	27 (84.4)	
Bone	19 (70.4)	25 (78.1)	
Abdominal/peritoneal	6 (22.2)	6 (18.8)	
Adrenal	0 (0)	2 (6.3)	
Brain	0 (0)	1 (3.1)	
Number of metastatic sites			
1	1 (3.7)	2 (6.3)	0.696
2	7 (25.9)	12 (37.5)	
3	12 (44.4)	11 (34.4)	
4	6 (22.2)	4 (12.5)	
5	1 (3.7)	2 (6.3)	
6	0	1 (3.1)	
ET sensitivity/resistance			
Sensitive or naive	13 (48.1)	6 (18.8)	0.035*
Primary resistance	4 (14.8)	4 (12.5)	
Secondary resistance	10 (37.0)	22 (68.8)	
Previous ET for ABC			
Yes	0 (0)	9 (28.1)	0.003*
No	27 (100)	23 (71.9)	
Organ in IVC/VC			
Liver	16 (59.3)	20 (62.5)	0.284
Lung/mediastinum	7 (25.9)	4 (12.5)	
Gastrointestinal	2 (7.4)	2 (6.3)	
Bone	1 (3.7)	2 (6.3)	
Multiple	1 (3.7)	4 (12.5)	
Liver + bone marrow	1	1	
Liver + lung	0	2	
Liver + lung + bone marrow	0	1	
IVC or VC			
IVC	16 (59.3)	8 (25.0)	0.009*
VC	11 (40.7)	24 (75.0)	

*p < 0.05

**Table 3:** Baseline blood test results of patients receiving CDK4/6i or paclitaxel

	CDK4/6i	Paclitaxel	*p* value
Full blood count (non-bone marrow IVC/VC)			
Haemoglobin (g/L)			
Median	133	123	0.100
Range	94–162	87–149	
Platelets (× 10^9^/L)			
Median	287	250	0.341
Range	114–610	39–737	
White cell count (× 10^9^/L)			
Median	9.3	8.0	0.209
Range	2.7–27.1	4–17.6	
Neutrophils (× 10^9^/L)			
Median	7	6.1	0.408
Range	0.8–21.2	2.2–19.7	
Other (all patients)			
Adjusted calcium (mmol/L)			
Median	2.44	2.36	0.190
Range	2.19–2.76	1.96–3.22	
LDH (IU/L)			
Median	407.5	656.0	0.010*
Range	214–1141	198–2504	
Hepatic profile (liver IVC/VC only)			
Albumin (µmol/L)			
Median	40	35	0.006*
Range	30–48	27–44	
Bilirubin (µmol/L)			
Median	10	17	0.151
Range	5–90	5–165	
AST/ALT (IU/L)			
Median	71	132	0.012*
Range	45–218	49–1640	
ALP (IU/L)			
Median	252	419	0.121
Range	63–814	95–1710	

*p < 0.05

### Treatment characteristics of patients with IVC/VC receiving CDK4/6i or paclitaxel

Table 4 shows treatment details of patients in both cohorts. The majority of patients in the CDK4/6i cohort received palbociclib (92.6%), and letrozole was the most common accompanying ET (85.2%). Dose reduction at cycle 1 was more frequent with paclitaxel compared to CDK4/6i and the most common reason was abnormal baseline hepatic profile. A similar proportion of patients in both cohorts had a dose reduction from cycle 2 onwards, and these were almost all due to treatment toxicities.

**Table 4:** Treatment characteristics of patients receiving CDK4/6i or paclitaxel

	CDK4/6i *n* = 27 (%)	Paclitaxel *n* = 32 (%)
Name of systemic therapy		
Palbociclib	25 (92.6)	–
Abemaciclib	2 (7.4)	–
Paclitaxel	–	30 (93.8)
Nab-paclitaxel	–	2 (6.2)
ET combined with CDK4/6i		
Letrozole	23 (85.2)	–
Fulvestrant	4 (14.8)	–
Ovarian suppression		
Yes	5 (18.5)	–
No	22 (81.5)	–
Dose reduction at baseline		
Yes	4 (14.8)	13 (40.6)
No	23 (85.2)	19 (59.4)
Reason for dose reduction at baseline		
Abnormal hepatic profile	2	9
Cytopenia	1	2
Performance status	1	1
Unknown	0	1
Dose reduction after cycle 1		
Yes	6 (22.2)	9 (28.1)
No	21 (77.8)	23 (71.9)
Reason for dose reduction after cycle 1		
Toxicity	5 (83.3)	9 (100)
Neutropenia	4	3
Cytopenia	1	2
Rash	0	1
Mucositis	0	1
Fatigue	0	1
Not recorded	0	1
Abnormal hepatic profile	1 (16.7)	0 (0)

### Treatment efficacy and outcomes of patients with IVC/VC receiving CDK4/6i vs. paclitaxel

Table 5 shows treatment efficacy and outcomes in both cohorts. Disease control rate at 4 months was numerically higher in the CDK4/6i compared to paclitaxel cohort, but did not reach statistical significance (77.8% vs. 59.4%, *p* = 0.168). Median time to first objective improvement in IVC/VC was similar in the CDK4/6i and paclitaxel cohorts (3.9 vs. 3.6 weeks, *p* = 0.773) and most patients showed an improvement within 4 weeks. Median time to first improvement was similar in patients with ET-sensitive vs. ET-resistant disease in both CDK4/6i (3.0 vs. 4.0 weeks, *p* = 0.518) and paclitaxel (3.4 vs. 4.3 weeks, *p* = 0.575) cohorts. Median time to first objective worsening in IVC/VC in patients that progressed by 4 months was not significantly different in CDK4/6i and paclitaxel cohorts (5.1 vs. 7.6 weeks, *p* = 0.356). The most common reason for stopping treatment in both cohorts was disease progression.

**Table 5:** Treatment efficacy and outcomes of patients receiving CDK4/6i or paclitaxel

	CDK4/6i (*n* = 27) (%)	Paclitaxel (*n* = 32) (%)	*p* value
Disease control at 4 months			
Response/stable disease	21 (77.8)	19 (59.4)	0.168
Progression	6 (22.2)	13 (40.6)	
Time to first objective improvement in IVC/VC (weeks) (in patients with disease control at 4 months)			
Median	3.9	3.6	0.773
Range	0.9–16	0.9–9.1	
≤ 4 weeks	14 (66.7)	11 (57.9)	0.353
4–8 weeks	4 (19.0)	7 (36.8)	
> 8 weeks	3 (14.3)	1 (5.3)	
Time to first objective worsening in IVC/VC (weeks) (in patients with disease progression by 4 months)			
Median	5.1	7.6	0.356
Range	1.7–10	0.3–16	
≤ 4 weeks	3 (50.0)	2 (15.4)	0.308
4–8 weeks	2 (33.3)	5 (38.5)	
> 8 weeks	1 (16.7)	6 (46.2)	
Reason for stopping treatment			
Disease progression	13 (48.1)	15 (46.9)	0.216
Death	3 (11.1)	3 (9.4)	
Toxicity	1 (3.7)	3 (9.4)	
Other	0 (0)	5 (15.6)	
Completed treatment	–	6 (18.8)	
N/A—Still on treatment	8 (29.6)	0 (0)	
Maintenance therapy on completion or stopping of paclitaxel			
Yes	–	11 (34.4)	
Aromatase inhibitor	–	4	
CDK4/6i + ET	–	3	
Fulvestrant	–	2	
Capecitabine	–	2	
No—persistent chemotherapy toxicity	–	1 (3.1)	
N/A—progression and/or death	–	20 (62.5)	
Second line treatment after progression (at any timepoint)			
Yes	14 (73.7)	12 (40.0)	0.039*
Chemotherapy	9	8	
CDK4/6i + ET	–	3	
Fulvestrant	3	1	
Aromatase inhibitor	1	–	
Exemestane + everolimus	1	–	
No—clinical deterioration and/or death	5 (26.3)	18 (60.0)	
N/A—not progressed on first line treatment	8	2	

*p < 0.05

11 (34.4%) patients in the paclitaxel cohort received maintenance treatment. The remaining majority of patients in the paclitaxel cohort did not receive maintenance treatment due to disease progression and/or death, and in one case treatment toxicity. Out of the 11 patients in the paclitaxel cohort who received maintenance treatment, three received maintenance CDK4/6i + ET. Of the remaining patients, one patient was not started on CDK4/6i due to concerns about liver dysfunction. The reasons for the other seven patients not having CDK4/6i as a maintenance treatment were not explicitly documented. However, two had been experiencing persistent toxicity from chemotherapy, one started capecitabine due to concerns about liver dysfunction and two had received previous single-agent ET for ABC which may have influenced oncologists’ views on further endocrine-based therapy.

A greater proportion of patients in the CDK4/6i cohort received second line treatment after progression (at any timepoint) (73.7% vs. 40.0%, *p* = 0.039). The reasons for patients not receiving second line treatment in both cohorts was either death from cancer and/or it not being safe to deliver further systemic therapy due to clinical deterioration secondary to cancer (except for one patient in the CDK4/6i cohort who died from drug-induced pneumonitis). Overall, 6 (18.8%) patients in the paclitaxel cohort received a CDK4/6i as a maintenance therapy or second line.

### Survival outcomes of patients with IVC/VC who received CDK4/6i

**Fig. 2:**
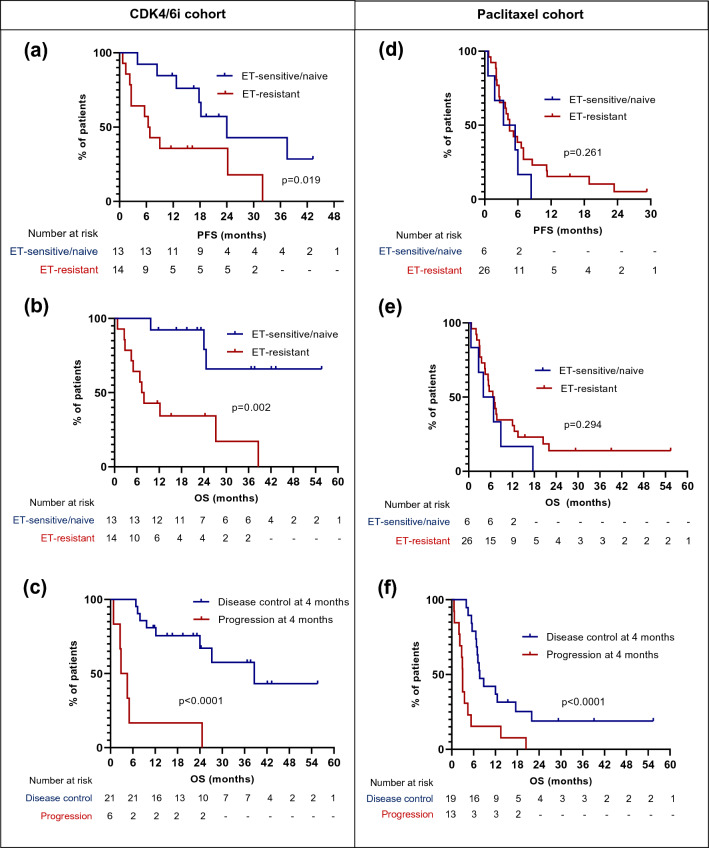
Progression-free survival (PFS) and overall survival (OS) in subgroups of patients with ER+/HER2- advanced breast cancer (ABC) and visceral crisis (VC) or impending VC (IVC) receiving first line treatment with CDK4/6i or weekly paclitaxel **a** CDK4/6i cohort: PFS in endocrine therapy (ET)-sensitive/naive vs. ET-resistant ABC; **b** CDK4/6i cohort: OS in ET-sensitive/naive vs. ET-resistant ABC; **c** CDK4/6i cohort: OS in patients with disease control vs. progression at 4 months after start of treatment **d** Paclitaxel cohort: PFS in ET-sensitive/naive vs. ET-resistant ABC; **e** Paclitaxel cohort: OS in ET-sensitive/naive vs. ET-resistant ABC; **f** Paclitaxel cohort: OS in patients with disease control vs. progression at 4 months after start of treatment

In the CDK4/6i cohort, at the study cut-off date, 13 (48.1%) patients had died due to cancer and 1 (3.7%) due to drug-induced pneumonitis and 13 (48.1%) were still alive. 8 (29.6%) were still receiving CDK4/6i treatment. The median TTF, PFS and OS were 17.3 months (95%CI 6.3–24.2), 17.8 months (95%CI 6.4–32.0) and 24.6 months (95%CI 7.8 months-not reached), respectively. As shown in Table 6, there was no significant difference in PFS or OS according to age, performance status, de novo vs. recurrent disease, number of metastatic sites, baseline LDH, dose reduction at baseline or presence of IVC vs. VC. Patients with ET-resistance had a shorter median PFS of 6.6 months compared to 24.0 months in patients with ET-sensitive/naive disease (*p* = 0.019) (Fig. 2a). Median OS was also shorter in patients with ET-resistant compared to ET-sensitive/naive cancer (7.6 months vs. not reached, *p* = 0.001) (Fig. 2b). Median OS was shorter in patients with liver compared to lung IVC/VC (18.1 months vs. not reached, *p* = 0.048). In patients with liver IVC/VC, there was no significant difference in PFS or OS according to degree of AST/ALT derangement (Table 6). Patients with disease control at 4 months had a median OS of 38.6 months compared to 3.7 months in those who had progressed by 4 months (HR 0.13, 95%CI 0.02–0.82, *p* < 0.0001) (Fig. 2c).

**Table 6:** Hazard ratios for PFS and OS in subgroups of patients receiving CDK4/6i

Factor	*n*	Hazard ratio—PFS (95% CI)	*p* value	Hazard ratio—OS (95% CI)	*p* value
Age					
< 65	17	1.00	0.643	1.00	0.553
≥ 65	10	0.87 (0.48–1.56)		0.72 (0.25–2.08)	
Performance status					
0	6	1.00		1.00	
1	18	1.22 (0.37–4.03)	0.095	0.80 (0.20–3.26)	0.745
≥ 2	3	2.24 (0.40–12.66)	0.307	3.86 (0.53–27.89)	0.065
De novo/recurrence					
De novo	11	1.00	0.359	1.00	0.267
Recurrence	16	0.66 (0.25–1.75)		0.58 (0.21–1.61)	
Number of metastatic sites					
1–2	8	1.00		1.00	
3	12	2.17 (0.71–6.60)	0.226	1.65 (0.47–5.81)	0.462
≥ 4	7	3.06 (0.72–13.03)	0.091	2.46 (0.52–11.63)	0.194
Baseline LDH (IU/L)					
< 500	15	1.00	0.901	1.00	0.774
≥ 500	7	0.93 (0.32–2.70)		1.19 (0.34–4.10)	
ET-sensitivity/resistance					
Sensitive or naive	13	1.00	0.019*	1.00	0.001*
Primary/secondary resistance	14	2.85 (1.09–7.48)		6.11 (2.07–18.06)	
Organ with IVC/VC					
Liver	16	1.00		1.00	
Lung	7	0.58 (0.21–1.56)	0.332	0.17 (0.05–0.53)	0.048*
Other or multiple	4	0.23 (0.07–0.78)	0.121	0.36 (0.09–1.46)	0.301
Dose reduction at baseline					
Yes	4	1.00	0.579	1.00	0.818
No	23	1.50 (0.42–5.33)		0.84 (0.17–4.14)	
IVC/VC					
IVC	16	1.00	0.291	1.00	0.257
VC	11	0.60 (0.23–1.55)		0.49 (0.16–1.46)	
Baseline AST/ALT level—liver IVC/VC					
1.2–2 × ULN	7	1.00		1.00	
2.1–3 × ULN	6	0.52 (0.16–1.71)	0.262	0.76 (0.22–2.63)	0.645
> 3 × ULN	4	0.73 (0.17–3.12)	0.674	1.10 (0.21–5.69)	0.899

*p < 0.05

### Survival outcomes of patients with IVC/VC who received paclitaxel

In the paclitaxel cohort, at the study cut-off date, 28 (87.5%) patients had died due to cancer and 4 (12.5%) were still alive. No patients were still on paclitaxel treatment but two were on first line maintenance treatment. The median TTF, PFS and OS were 3.5 months (95%CI 2.4–4.5), 4.5 months (95%CI 2.5–6.4), and 6.7 months (95%CI 4.0–7.8), respectively. As shown in Table 7, there was no significant difference in PFS or OS according to age, performance status, de novo/recurrence, number of metastatic sites, baseline LDH, organ affected by IVC/VC, dose reduction at baseline, presence of IVC vs. VC, previous ET for ABC, degree of AST/ALT derangement in liver IVC/VC or ET-sensitive/naive vs. ET-resistant ABC (Fig. 2d, e). Patients with disease control at 4 months after starting treatment had a longer median OS of 7.7 months compared to 3.0 months in patients who had progression (HR 0.30, 95%CI 0.12–0.75, p < 0.0001) (Fig. 2f). Patients who received a CDK4/6i as maintenance treatment or second line after paclitaxel had a longer median PFS (18.9 months vs. 4.1 months, *p* = 0.0005) and OS (not reached vs. 5.4 months, *p* = 0.0003) compared to those that did not.

**Table 7:** Hazard ratios for PFS and OS in subgroups of patients receiving paclitaxel

Factor	*n*	Hazard ratio—PFS (95% CI)	*p* value	Hazard ratio—OS (95% CI)	*p* value
Age					
< 65	10	1.00	0.834	1.00	0.592
≥ 65	22	1.08 (0.50–2.34)		1.23 (0.55–2.74)	
Performance status					
0–1	17	1.00	0.455	1.00	0.808
≥ 2	15	0.75 (0.33–1.66)		0.89 (0.36–2.20)	
De novo/recurrence					
De novo	2	1.00	0.232	1.00	0.150
Recurrence	30	0.43 (0.05–3.53)		0.37 (0.04–3.54)	
Number of metastatic sites					
1–2	14	1.00		1.00	
3	11	1.36 (0.60–3.11)	0.415	1.96 (0.79–4.88)	0.098
≥ 4	7	1.03 (0.39–2.73)	0.950	1.06 (0.39–2.86)	0.905
Baseline LDH (IU/L)					
< 500	6	1.00	0.364	1.00	0.384
≥ 500	12	1.58 (0.60–4.17)		1.57 (0.58–4.23)	
ET-sensitivity/resistance					
Sensitive or naive	6	1.00	0.261	1.00	0.294
Primary or secondary resistance	26	0.61 (0.21–1.75)		0.62 (0.22–1.78)	
Organ with IVC/VC					
Liver	20	1.00		1.00	
Lung	4	1.72 (0.46–6.34)	0.306	2.07 (0.54–7.98)	0.291
Other or multiple	8	0.72 (0.31–1.66)	0.460	0.79 (0.33–1.91)	0.620
Dose reduction at baseline					
Yes	13	1.00	0.283	1.00	0.231
No	19	0.68 (0.32–1.44)		0.64 (0.29–1.41)	
IVC/VC					
IVC	8	1.00	0.773	1.00	0.751
VC	24	0.87 (0.33–2.27)		0.87 (0.36–2.12)	
Baseline AST/ALT level—liver IVC/VC					
1.2–2 × ULN	4	1.00	0.330	1.00	0.755
2.1–3 × ULN	6	0.57 (0.14–2.29)		0.93 (0.25–3.47)	
3–6 × ULN	7	0.58 (0.15–2.28)	0.329	0.83 (0.23–2.92)	0.823
≥ 6 × ULN	7	0.81 (0.23- 2.89)	0.852	0.58 (0.15–2.32)	0.365
Previous ET for ABC					
No	23	1.00	0.984	1.00	0.269
Yes	9	1.28 (0.56–2.93)		1.55 (0.65–3.71)	
CDK4/6i as maintenance therapy or second line after paclitaxel					
No	26	1.00	0.0005*	1.00	0.0003*
Yes	6	0.23 (0.11–0.47)		0.23 (0.06–0.26)	

*p < 0.05

### Comparison of survival outcomes in patients with IVC/VC treated with CDK4/6i vs. paclitaxel

**Fig. 3:**
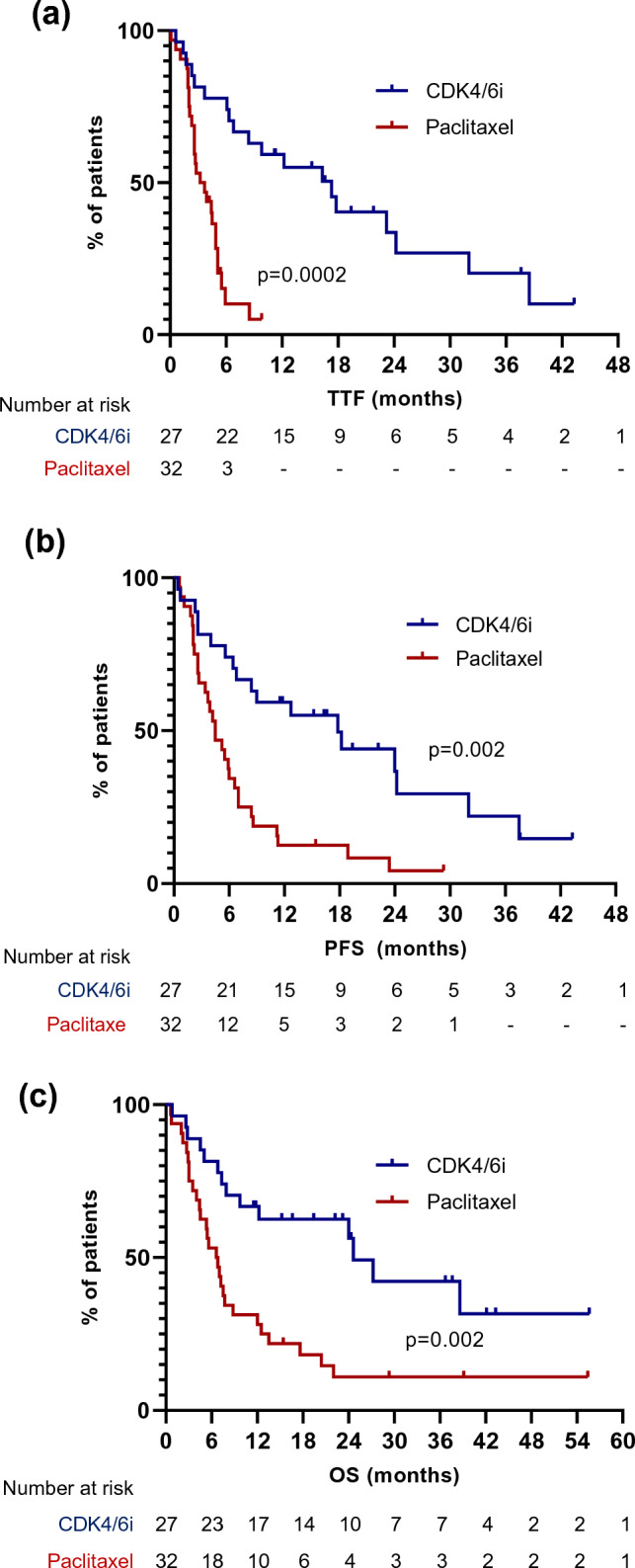
Outcomes of patients receiving CDK4/6i compared to weekly paclitaxel for ER+/HER2- advanced breast cancer with visceral crisis (VC) or impending VC. **a** Time to treatment failure (TTF); **b** Progression-free survival (PFS); **c** Overall survival (OS)

Median TTF, PFS, and OS were all significantly longer in patients who were treated with CDK4/6i compared to paclitaxel – TTF 17.3 vs. 3.5 months (HR 0.33, 95%CI 0.17–0.61, *p* = 0.0002), PFS 17.8 vs. 4.5 months (HR 0.38, 95%CI 0.21–0.67, *p* = 0.002), OS 24.6 vs. 6.7 months (HR 0.37, 95%CI 0.20–0.68, *p* = 0.002) (Fig. 3a–c). Table 8 shows that differences in survival were maintained in the majority of subgroups. However, in patients with ET-resistance, there was no significant difference in PFS or OS between the two cohorts. In multivariate analysis, when considering organ with IVC/VC (liver-only vs. lung-only vs. other), ET-resistance vs. ET-sensitivity, presence of IVC vs. VC, de novo vs. recurrent disease and PS (0–1 vs. ≥ 2) as covariables, treatment with CDK4/6i was independently predictive of a longer PFS (HR 0.31, 95%CI 0.12–0.78, *p* = 0.015). Patients receiving CDK4/6i also had a longer OS in multivariate analysis but this did not quite reach statistical significance (HR 0.41, 95%CI 0.16–1.03, *p* = 0.062), although PS ≥ 2 was significantly predictive of shorter OS (HR 2.39, 95%CI 1.13–5.03, *p* = 0.021).

**Table 8:** Hazard ratios for PFS and OS in patients who received CDK4/6i vs. paclitaxel stratified by different subgroups

Factor	*n*	Hazard ratio—PFS (95% CI)	*p* value	Hazard ratio—OS (95% CI)	*p* value
VC					
CDK4/6i	11	1.00	0.019*	1.00	0.014*
Paclitaxel	24	2.73 (1.29–5.77)		3.48 (1.56–7.75)	
IVC					
CDK4/6i	16	1.00	0.007*	1.00	0.027*
Paclitaxel	8	3.03 (0.97–9.46)		2.38 (0.77–7.40)	
ET-sensitive/naive					
CDK4/6i	13	1.00	< 0.0001*	1.00	< 0.0001*
Paclitaxel	6	6.51 (1.20–35.45)		11.72 (1.84–74.64)	
Primary/secondary ET-resistance					
CDK4/6i	14	1.00	0.199	1.00	0.653
Paclitaxel	26	1.56 (0.80–3.05)		1.18 (0.58–2.39)	
All patients—excluding previous ET for ABC					
CDK4/6i	27	1.00	0.003*	1.00	0.011*
Paclitaxel	23	2.45 (1.25–4.79)		2.36 (1.16–4.80)	
Liver only IVC/VC					
CDK4/6i	16	1.00	0.015*	1.00	0.096
Paclitaxel	20	2.21 (1.11–4.43)		1.84 (0.88–3.83)	
Lung only IVC/VC					
CDK4/6i	7	1.00	0.012*	1.00	0.009*
Paclitaxel	4	4.37 (0.74–25.91)		10.15 (1.45–71.07)	

*p < 0.05

## Discussion

The optimal treatment for ER+/HER2- ABC with IVC/VC is unclear and chemotherapy is often given instead of CDK4/6i based on the theory it may be more rapidly efficacious and have better survival outcomes. However, there is limited evidence to confirm which treatment is superior. Weekly paclitaxel is a chemotherapy regimen commonly used in the setting of IVC/VC at our institution due to the ability to deliver more safely and dose adjust and monitor weekly. This is the first known detailed retrospective study comparing efficacy and outcomes of patients with ER+/HER2- ABC and IVC/VC treated with a CDK4/6i + ET or weekly paclitaxel first line.

In this study, the frequency of IVC/VC in patients with ER+/HER2- ABC receiving first line CDK4/6i or paclitaxel was 12.2%, which is similar to previous reported rates [6, 10, 11]. The liver was the most common organ of IVC/VC in both cohorts. Patients treated with CDK4/6i had a significantly longer TTF, PFS and OS compared to paclitaxel. This agrees with two previous retrospective studies showing patients with ER+/HER2- ABC and VC treated with a CDK4/6i + ET had a longer OS compared to patients receiving other systemic therapies, including chemotherapy [10, 11]. The survival outcomes of the paclitaxel cohort are also comparable to a retrospective study of patients with ABC and VC (80% ER+) who received weekly paclitaxel + bevacizumab [9]. In our study, patients in the paclitaxel cohort who received a CDK4/6i as maintenance or second line had a longer OS and PFS compared to patients who did not, suggesting CDK4/6i use leads to better survival outcomes even if not used first line. OS was significantly worse in patients with liver only IVC/VC. A retrospective study of patients with ABC and VC, including ER+/HER2- cases, also showed worse prognosis in patients with liver VC compared to other organ(s) of VC [11].

Patients who received paclitaxel had a higher incidence of recurrent disease, secondary ET-resistance and/or VC and a lower serum albumin, higher LDH, and higher AST/ALT (liver IVC/VC only) at baseline. This suggests a higher degree of baseline organ dysfunction and ET-resistance in the paclitaxel compared to CDK4/6i cohort, which may at least partly be due to these factors increasing the likelihood of chemotherapy being given. In both cohorts, there was no significant difference in OS or PFS when stratifying by VC vs. IVC, or by degree of AST/ALT derangement in liver only IVC/VC. Therefore, severity of organ dysfunction based on these parameters may not be predictive of disease control or survival in patients receiving these treatments. Performance status was overall worse in the paclitaxel cohort although differences in PS were not significantly predictive of PFS or OS in univariate analysis in either the CDK4/6i or paclitaxel cohorts. However, in multivariate analysis PS ≥ 2 was significantly predictive of shorter OS, suggesting that PS is an important prognostic factor in these patients. More patients in the paclitaxel cohort had a dose reduction from cycle 1, which in most was due to an abnormal hepatic profile. In theory, dose reduction could lead to reduced efficacy, but in this study, it was not predictive of PFS or OS. The time to first objective improvement in IVC/VC was similar in both cohorts, suggesting a similar speed of reversal in organ dysfunction, and was not significantly different in patients with ET-sensitive vs. ET-resistant disease. The majority of patients who had disease control at 4 months showed an objective improvement within 4 weeks of starting treatment—67% of the CDK4/6i and 58% of the paclitaxel cohort. Disease control at 4 months in both cohorts was predictive of much better OS. Although patients receiving CDK4/6i had a numerically higher disease control rate at 4 months than those receiving paclitaxel, this did not reach statistical significance.

Patients who received a first line CDK4/6i maintained a longer PFS and OS compared to paclitaxel in the majority of subgroup analyses. Patients with ET-sensitive/naive ABC had a longer PFS and OS in the CDK4/6i cohort, whereas those with ET-resistance had a similar PFS and OS in both cohorts. This suggests CDK4/6i could be considered as a first line treatment for both ET-sensitive and ET-resistant ABC with IVC/VC, but is particularly beneficial in ET-sensitive/naive ABC. Use of CDK4/6i first line was independently predictive of longer PFS compared to paclitaxel when considering organ with IVC/VC, ET-resistance vs. ET-sensitivity, IVC vs. VC, de novo vs. recurrent disease and PS as covariables.

The strengths of this study are that it was carried out at a large comprehensive tertiary cancer centre with over 1000 cases screened to identify eligible patients, and included patients with IVC as well as VC. Baseline characteristics including age, number of metastases, organ of IVC/VC were also not significantly different between the CDK4/6i and paclitaxel cohorts. The limitations of this study are that data were collected retrospectively from a single centre and tumours could not all be assessed to formal RECIST criteria. The paclitaxel cohort also had more ET-resistance, more recurrent disease, more VC compared to IVC, worse PS and worse baseline hepatic profile (in liver IVC/VC) compared to the CDK4/6i cohort. The presence of these characteristics may have increased the likelihood of oncologists choosing paclitaxel rather than CDK4/6i as first line treatment. These characteristics are associated with a poorer prognosis in ABC and may have resulted in a selection bias if oncologists were less keen to treat patients with extensive disease and poorer PS with a relatively novel oral therapy. This represents a weakness in the study that is likely to have affected the results, even though these characteristics were not individually predictive of PFS or OS, and in multivariate analysis which included these characteristics as co-variables, use of CDK4/6i first line was independently predictive of longer PFS. No patients in the CDK4/6i cohort had an AST/ALT > 6 × ULN so it was not possible to compare outcomes in patients at the most extreme end of liver dysfunction. The use of single-agent rather than doublet chemotherapy in this study may also have limited potential efficacy, although the paclitaxel cohort had a better median OS of 6.7 months compared to 3.7 months in a previous study of patients with VC who received platinum combination chemotherapy [8].

## Conclusion

Patients with ER+/HER2- ABC and IVC/VC treated first line with a CDK4/6i had a significantly better survival and similar speed of improvement in IVC/VC compared to patients treated with weekly paclitaxel. This suggests CDK4/6i should be considered as a first line treatment option for patients with IVC/VC. Further prospective studies are recommended to confirm these results, and at least one is currently underway.

## Data Availability

The datasets generated and/or analysed during this study are not publicly available due to patient confidentiality but are available from the corresponding author upon reasonable request.
